# Burnout and quality of life among correctional officers in a women’s correctional facility

**DOI:** 10.47626/1679-4435-2020-561

**Published:** 2021-02-11

**Authors:** Lidiany da Silva Venâncio, Bernardo Diniz Coutinho, Daniela Gardano Bucharles Mont’Alverne, Rodrigo Fragoso Andrade

**Affiliations:** Departamento de Fisioterapia, Universidade Federal do Ceará - Fortaleza (CE), Brazil

**Keywords:** burnout, quality of life, occupational health, correctional facility

## Abstract

**Introduction:**

The correctional officer career is considered a stressful and risky occupation that can affect the mental health of workers due to stress and burnout; this syndrome presents itself with physical, psychological, behavioral, and defensive symptoms, ultimately affecting quality of life.

**Objectives:**

To evaluate the sociodemographic profile, burnout levels, and quality of life of female correctional officers in a women’s correctional facility, as well as to verify possible correlations between these variables.

**Methods:**

This is a descriptive cross-sectional study performed in a women’s correctional facility located in Aquiraz, in the state of Ceará, through the use of 3 evaluation instruments: the Maslach Burnout Inventory-General Survey, the abbreviated version of the World Health Organization Quality of Life instrument, and a general information questionnaire.

**Results:**

Most of the correctional officers were married or cohabiting, aged between 31 and 40 years old, with complete or partial undergraduate education, and at least 1 child. In the burnout investigation, mean scores were 1.9±1.43, indicating a moderate level of burnout. Regarding quality of life, the environment domain presented the lowest scores (57.34%). We observed a correlation between burnout and quality of life, in which the higher the burnout scores, the lower the quality of life reported by correctional officers.

**Conclusions:**

Our data demonstrate that correctional officers face a risk of developing burnout, thus affecting their quality of life; therefore, preventive health care measures are required for these professionals.

## INTRODUCTION

The correctional officer career is considered risky and stressful, and can result in both physical and psychological disorders since risk and vulnerability are intrinsic to this occupation. These professionals are in direct contact with incarcerated offenders and are responsible for their surveillance, custody, and discipline; in addition, correctional officers contribute to the social reintegration of inmates, thus preventing recidivism.^1^

The current situation in Brazilian correctional facilities does not provide adequate conditions for officers to adequately perform their tasks due to prison overcrowding, a small number of professionals, and work overload. These situations result in poor working conditions, which associated to the feeling of being undervalued and professionally frustrated can affect quality of life, as well as the physical and mental health of these workers.^2,4^

Approximately 37% of correctional officers may present symptoms characteristic of burnout.^5^ This syndrome is defined as a chronic psychological phenomenon present in individuals whose work requires intense and frequent attention, as well as a contact with people that require assistance and care.^6,7^

Burnout comprises 3 main characteristics that may or may not be associated: emotional exhaustion (EE), regarding the depletion of physical and emotional energy; cynicism (CY), considering indifference and emotionally distant attitudes towards work; and work self-efficacy (SE), which is related to the expectations at work.^8^

Symptoms associated with burnout can be physical (sleep disorders, constant and progressive fatigue, muscle pain, headaches, and gastrointestinal, cardiovascular, respiratory, or sexual disorders) or psychological (lack of attention and concentration, solitude, impatience, low self-esteem, discouragement, and depression). Behavioral symptoms can also be present (inability to relax, irritability, aggressiveness, or even high-risk behaviors and suicide), as well as defensive symptoms (isolation, lack of interest on work or leisure, irony, and CY).^9^

Burnout symptoms in male correctional officers are known to be expressed though lack of motivation, thus generating unproductive attitudes and behaviors and resulting not only on a lack of commitment to the job, but also in reduced facility security and inmate social reintegration.^10^ However, studies on this subject considering female correctional officers are rare.

Diseases related to chronic stress - such as burnout - can directly influence the execution of work tasks, thus affecting the quality of life of workers.^11^ The concept of quality of life is multidimensional, complex, and dynamic, in addition to being specific for each individual according to the environment and context in which one is located. Similar conditions may result in different perceptions of quality of life. In order for a person to have good health, quality of life is a pre-requisite, and not the other way around.^12^

The World Health Organization (WHO) defines quality of life as an individual’s perception of his or her position in life in the context of culture and values and regarding his or her objectives, expectations, standards, and concerns. Quality of life thus comprises physical health, psychological state, and social relationships, among other aspects of life.^13^

Evaluating of quality of life has become increasingly important in occupational health studies that aim to promote care strategies for improving the mental and physical health of the population. When related to work, quality of life is directly connected to the satisfaction, mental health, expectations, wishes, and pleasures of an individual with regards to the work environment, in addition to a desire to be perceived as a part of this environment. Therefore, quality of life is a subjective dimension whose evaluation is extremely important in the identification of risks to workers’ mental health, since quality of life relates to various areas of life such as work, family, and leisure.^14^

Considering the highly stressful environment in which correctional officers work, the low visibility of strategies that promote the physical and mental health of these professionals, and the lack of studies regarding burnout in this population, in this study we aimed to evaluate the sociodemographic profile, as well as the levels of burnout and quality of life and their possible correlations in female correctional officers working in a women’s correctional facility.

## METHODS

This is a descriptive cross-sectional study that investigated burnout symptoms and aspects related to quality of life in correctional officers working in a women’s correctional facility located in Aquiraz, in the state of Ceará. This study was approved by the Ethics and Research Committee (decision No. 1733917) according to Resolution 466/2012 of the National Health Council.

### PARTICIPANTS

We initially obtained the total number of correctional officers working in the facility and then presented the objectives and methods to be used in this research to the potential participants. Out of 50 officers, 40 showed interest in participating in our study and were instructed to sign the free and informed consent form (FICF).

### EXCLUSION CRITERIA

We excluded from this study agents that refused to sign the FICF and those that were on leave (for any reason) during the period of interviews.

### DATA COLLECTION

Our data collection was performed by a trained female interviewer using the evaluation instruments; in-person individual meetings happened in a private room, favoring the wellbeing and privacy of participants. Data collection was performed during the officers’ working hours.

### EVALUATION INSTRUMENTS

The evaluations were performed through the use of 3 specific instruments: a sociodemographic, professional, and health profile questionnaire, a quality of life questionnaire, and a burnout symptom assessment. The first questionnaire was elaborated by the authors and assessed sociodemographic variables (age, sex, marital status, education, children, household situation, and form of transportation); professional variables (duration of employment, working hours, overtime, position, occupation, reason for choosing the profession); and health condition variables (regular sport/physical exercise, diseases, daily hours of sleep, sleep quality, and drinking habits).

Quality of life was assessed using the abbreviated version of the WHO Quality of Life (WHOQOL-Bref) instrument, which was translated and validated by the WHO research group on quality of life in Brazil. WHOQOL-Bref comprises 4 domains: physical, psychological, social relationships, and environment. A reference value of 100% indicates the highest possible level of quality of life.^15^

For evaluating burnout symptoms, we used the Maslach Burnout Inventory - General Survey (MBI-GS) questionnaire, developed in 1981 by Maslach and Jackson and since then used in various occupational groups.^7^ The MBI-GS consists of 16 questions divided into 3 dimensions: EE (6 items), CY (4 items), and SE (6 items). Burnout levels are measured using scores, which are considered low when below 1.33, intermediate when between 1.34 and 2.43, and high when above 2.43 (according to McLaurine).^16^ This classification further specifies the scores dividing them by dimension (Table 1).

**Table 1:** Scores used for assessment of burnout levels (general and for each dimension).

	Low	Moderate	High
Burnout (general)	< 1.33	1.34-2.43	> 2.43
Emotional exhaustion	< 2.00	2.10-3.19	> 3.20
Cynicism	< 1.00	1.01-2.10	> 2.20
Work self-efficacy	< 4.00	4.01-4.99	> 5.00

Source: McLaurine.^16^

As instructed by Schuster et al.,^8^ results were calculated through the sum of the results obtained for each dimension divided by the number of items of that dimension; this resulted in 3 weighted means. The SE dimension has an inverse relationship with the scores when compared to EE and CY, thus its calculation was performed after inversing the scores for each answer (0 was switched to 6, 1 was switched to 5, and so on). This way, we obtained a reduced SE index (rSE).

### DATA ANALYSIS

The obtained data were analyzed independently through descriptive statistics and were recorded on Microsoft Excel 2007 spreadsheets; the sociodemographic, professional, and quality of life results were described through percentages, while burnout levels were reported as means and standard deviations (SD). SPSS software version 17.0 was used for verifying correlations between burnout and quality of life through the Pearson correlation index; p-values ≤ 0.05 were considered statistically significant.

## RESULTS

Most of the interviewed correctional officers were married or cohabiting (66%), aged between 31 and 40 years (58%), had complete or partial undergraduate education (68%), at least 1 child (61%), and lived with their families (83%), as shown in Table 2.

**Table 2:** Sociodemographic, professional, and health condition variables regarding correctional officers in a women's correctional facility in the state of Ceará.

Variables	n (%)
Sociodemographic	
Marital status	
Single	9 (23)
Married/cohabiting	26 (66)
Divorced	3 (8)
Widow	2 (5)
Age (years)	
20-30	10 (26)
31-40	23 (58)
> 41	7 (18)
Education	
Secondary education	13 (33)
Partial undergraduate education	11 (28)
Complete undergraduate education	16 (40)
Children	
None	16 (40)
1-2	23 (58)
3 or more	1 (3)
Lives with:	
Family	33 (83)
Friends	1 (3)
Alone	2 (5)
Others	4 (10)
Professional	
Occupation	
Operational	32 (80)
Reception	4 (10)
Director	4 (10)
Overtime?	
Yes	30 (75)
No	10 (25)
Weekly overtime (hours)	
Less than 12	22 (81)
12-24	4 (15)
More than 24	1 (4)
Duration of employment (years)	
1-3	22 (55)
3-6	17 (42)
6-10	1 (3)
More than 10	0 (0)
Why did you choose this profession?	
Aptitude	6 (15)
Financial reasons	24 (60)
Other reasons	10 (25)
Health conditions	
Regular physical activity	
Yes	20 (50)
No	20 (50)
Diseases	
Yes	5 (13)
No	35 (88)
Daily hours of sleep	
2-4	1 (3)
4-6	12 (30)
6-8	17 (43)
More than 8	10 (25)
Sleep quality	
Poor/very poor	4 (10)
Average	10 (25)
Good	11 (28)
Great/excellent	15 (38)
Drinking habits	
Always	0 (0)
Sometimes	9 (23)
Rarely	18 (45)
Never	13 (33)

Out of 40 correctional officers, 32 worked operational jobs that required general surveillance, prisoner inspection and escort, as well as promoting the safety of inmates and the facility. The weekly workload for all officers was 48 hours, in 24-hour shifts separated by 72 hours off; however, most of the interviewed officers (75%) reported working overtime (up to 12 hours a week). Regarding the duration of their employment at the women’s correctional facility, 97% had worked at their positions for 1 to 6 years. When questioned about the reasons for choosing this profession, most of the participants mentioned financial reasons (60%), while a small percentage (15%) reported an aptitude for this occupation.

Half of the participants had regular physical activity, and most of them (88%) did not report any type of disease. Regarding daily hours of sleep, 68% reported 6 or more hours of sleep per day, while 66% answered that sleep quality was at least good. Finally, most of the participants (78%) reported rarely or never consuming alcohol.

**Figure 1:**
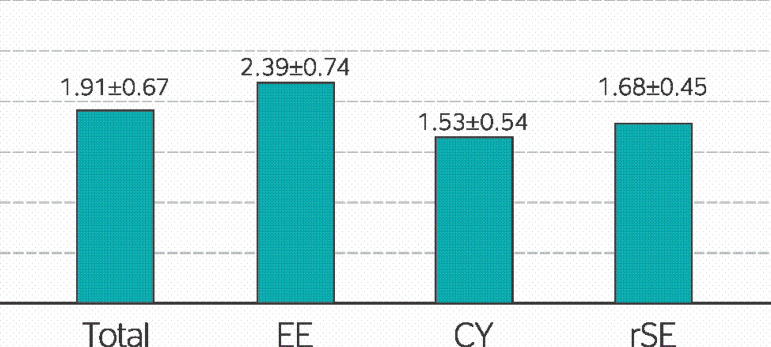
Distribution of weighted means in the Maslach Burnout Inventory-General Survey (MBI-GS) scale. Investigation of burnout among correctional officers in a women’s correctional facility in the state of Ceará. Results are expressed as means and standard deviation (SD). Total: general scores obtained in the burnout questionnaire; EE: emotional exhaustion; CY: cynicism; rSE: reduced work self-efficacy.

The results of the MBI-GS questionnaire achieved mean values of 1.9 (SD, 1.43), indicating that the sample had a moderate burnout level. The EE dimension presented mean results of 2.39 (SD, 1.59), representative of a moderate level of exhaustion. Mean results for the CY dimension were 1.53 (SD, 1.83), also representing a moderate level. Finally, the mean rSE results were 1.68 (SD, 1.64), indicating a low burnout level in this dimension. Figure 1 illustrates the results obtained in the general burnout evaluation and in each dimension of the MBI-GS.

**Figure 2:**
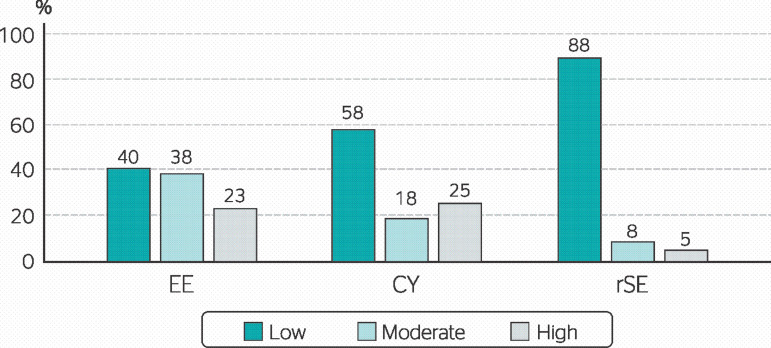
Detailed distribution of results for each dimension of the Maslach Burnout Inventory-General Survey (MBI-GS) among correctional officers in a women’s correctional facility in the state of Ceará. EE: emotional exhaustion; CY: cynicism; rSE: reduced work self-efficacy.

A more detailed analysis of each dimension indicated that EE and CY showed higher percentages of moderate and high burnout levels (61% and 43%, respectively), while rSE presented only 13% (Figure 2).

**Figure 3:**
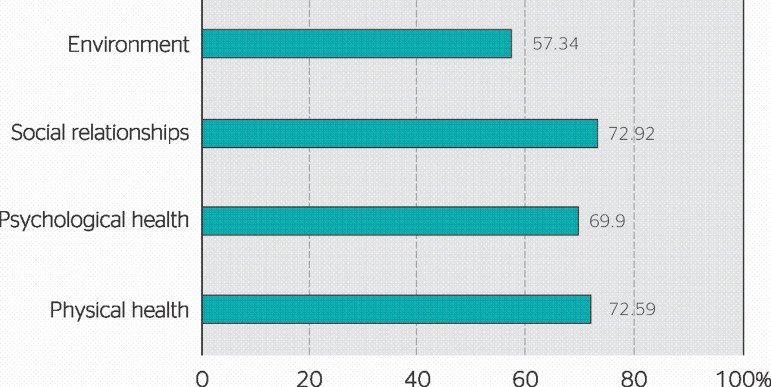
Results for each domain of the abbreviated version of the World Health Organization Quality of Life instrument (WHOQOL-Bref) regarding correctional officers in a women’s correctional facility in the state of Ceará.

Regarding quality of life, the domains comprising physical health (72.59%) and social relationships (72.92%) presented the best mean results, followed by the psychological health (69.90%) and environment (57.34%) domains (Figure 3).

**Figure 4:**
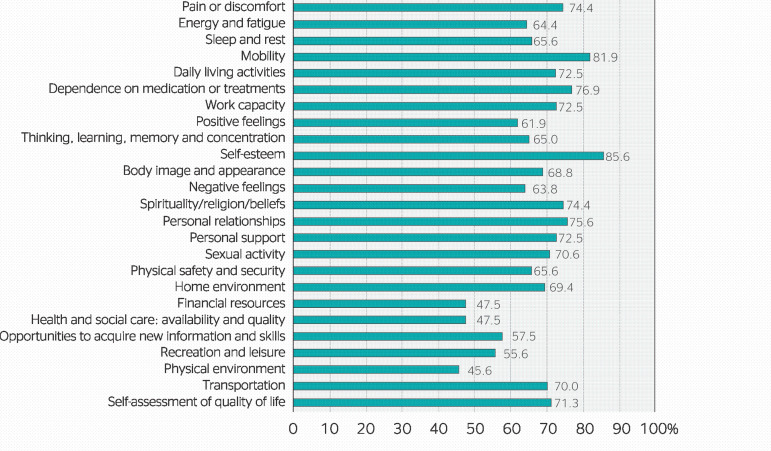
Results for each item of the abbreviated version of the World Health Organization Quality of Life instrument (WHOQOL-Bref) regarding correctional officers in a women’s correctional facility in the state of Ceará.

Within the physical health domain, the highest results were obtained for mobility (81.88%) and the lowest, for energy and fatigue (64.38%) as well as sleep and rest (65.63%). In the psychological health domain, self-esteem presented the highest result among all domains (85.63%), while positive feelings accounted for 61.88%. All items of the social relationships domain presented results above 70%. The environment domain had the lowest scores, and financial resources and health care were the items with the lowest scores among all domains (both with 47.50%). Figure 4 illustrates the results of all items of the WHOQOL-Bref instrument.

We did not find significant correlations between burnout and any of the following variables: education, occupation, reason for choosing the profession, and regular physical activity. However, we observed that older correctional officers presented lower levels of burnout (EE, r = -0.314; p = 0.049; CY, r = -0.193, p = 0.233; rSE, r = -0.452, p = 0.003).

Among each of the burnout dimensions, we verified that higher EE scores were related to higher CY (r = 0.785; p = 0.000) and rSE (r = 0.491; p = 0.001) scores, and higher CY scores were related to higher rSE (r = 0.566; p = 0.000). When evaluating possible correlations between each dimension of the MBI-GS instrument and the WHOQOL-Bref domains, we observed that the higher the scores for each MBI-GS dimension, the lower the quality of life in the physical and psychological health domains, while personal relationships and environment were not significantly correlated (Table 3).

**Table 3:** Correlations between burnout levels (Maslach Burnout Inventory-General Survey [MBI-GS]) and quality of life (World Health Organization Quality of Life instrument-abbreviated version [WHOQOL-Bref]) among correctional officers in a women's correctional facility in the state of Ceará.

Burnout dimension	D1	D2	D3	D4
Emotional exhaustion	r = -0.567; p = 0.000*	r = -0.386; p = 0.014*	r = -0.117; p = 0.472	r = -0.227; p = 0.160
Cynicism	r = -0.483; p = 0.002*	r = -0.472; p = 0.002*	r = -0.271; p = 0.091	r = -0.282; p = 0.078
Work self-efficacy	r = -0.210; p = 0.194	r = -0.406; p = 0.009*	r = -0.190; p = 0.239	r = -0.094; p = 0.566

D1: physical health domain; D2: psychological health domain; D3: personal relationships domain; D4: environment domain.

* p ≤ 0.05.

## DISCUSSION

Our results demonstrate that more than half of the correctional officers were married or cohabiting, aged between 31 and 40 years, and had complete or partial undergraduate education, with at least 1 child. Similar results were reported by Mayer et al.^17^ among 240 military police officers in Campo Grande, state of Mato Grosso do Sul: Analyzing burnout levels and professional quality of life, the researchers verified that their sample was aged between 25 and 40 years, had up to 3 children, was predominantly married and had complete secondary education. This study did not find a correlation between age and burnout, which differs from our results: among the participants of our study, the older the correctional officers were, the lower were burnout levels.

Mayer et al.^17^ also highlighted that education could influence the participants’ perception of their satisfaction with organizational inter-relations, making them more or less demanding. This was not observed in our study, since there were no correlations between schooling and burnout levels.

In recent years, the schooling level of correctional officers has presented some changes; new professionals are currently in their undergraduate studies or already have an undergraduate degree in areas such as law, psychology, and social services. More qualified candidates are currently seeking this profession, and the reasons for entering this career path, as reported by correctional officers, include not only an aptitude for a career in security, but also a lack of other job opportunities, job stability, the opportunity of working in the public sector, and the expectation of achieving other occupations.^2,4^ In this study, we observed similar results, since 68% of the participants had complete or partial undergraduate education and only 15% of them reported having chosen this career owing to an aptitude; most of the interviewed officers reported financial reasons.

Some officers also highlighted the fact of working in shifts, with satisfactory off days, as a reason for choosing this career: 24-hour shifts are followed by 72 hours off work. However, 75% of them also reported working overtime due to financial reasons, which could contribute to overwork complaints and expose the workers to longer hours of the environmental risks of a knowingly stressful and dangerous workplace.^4^

When evaluating the different occupations within the correctional facility, there were no differences between officers that performed operational work, those working in the reception, and those that had director jobs: Regardless of their occupation, the risk of developing burnout was the same. These data differ from other studies, which reported that correctional officers on operational jobs had higher stress and burnout levels in comparison to those working supervisor jobs or those who did not work in custody.^18,19^

A systematic review performed in 2013 by Finney et al.^20^ evaluated the organizational stressors related to work stress and burnout among correctional officers and observed that occupation and other stressors such as overtime and prison overcrowding had inconsistent results regarding the development of burnout. However, the organizational structure and workplace environment were directly associated with stress and burnout in these professionals.

Our results corroborated those reported by the Maslach e Jackson^21^ MBI manual, which reported that an individual experiencing burnout had elevated EE and CY levels and low rSE levels. More than half of our sample (60%) presented moderate to high levels of burnout in the EE dimension, revealing that correctional officers faced physical or emotional exhaustion, mainly caused by an overload of tasks and interpersonal conflicts. This can be explained by the fact that officers have a direct contact with inmates, are constantly aware of their problems, and are held accountable for the difficulties faced by prisoners such as prison overcrowding and a lack of infrastructure and health care. These aspects result in an emotional overload on the correctional officers.

Moreover, the workload inside a correctional facility is known as a strong stressor factor, since the officers need to handle health, social, justice, and prisoner escort services, as well as supervise the meal times and locking of cells. These activities, when associated to prison overcrowding and a reduced number of officers, result in the physical overload of these professionals.^2^

Within the CY dimension, moderate to high burnout levels were shown by 43% of our sample, which revealed that officers develop insensitive attitudes in the relationship with people at work, frequently acting with insensitivity and severity; this is a typical dimension of burnout and differentiates it from work-related stress.^22^ These types of attitudes may represent an attempt by the officer to defend herself from the emotional load of the direct contact with others, with the aim of creating a barrier that prevents other people’s suffering and problems from affecting their lives.^23^

Considering the rSE domain, 88% of the correctional officers interviewed in this study presented low burnout levels, revealing that they did not feel unsatisfied or incompetent at work. These data are in line with those reported by Santos and Santos^24^ considering quality of life at work in the State Correctional Facility of Ponta Grossa, state of Paraná. Out of 61 correctional officers, 73% stated that their work was important for their professional realization and that they were happy with their tasks and the relevance of their work. Our data also corroborated the study by Tschiedel and Monteiro,^25^ which analyzed female correctional officers in the state of Rio Grande do Sul and reported that the participants demonstrated contentment with their wages and work stability, in addition to reporting pleasure in performing their labor.

As a strategy for facing burnout symptoms, Satler^3^ recommends regular physical exercise. However, in this study, we did not find a significant correlation between burnout and physical activities.

When it comes to quality of life, the environment domain presented the lower scores; this could be due to the fact that correctional officers are exposed to risks that are inherent to this profession, such as violence and the exposure to biological agents.^2,26^ These results are in accordance with Fernandes et al.,^26^ who evaluated quality of life and stress among correctional officers in the state of Paraíba and also reported that the environment domain received the lowest scores. This domain comprises aspects such as physical safety, financial resources, health care, household environment, recreation and leisure, and transportation.

The physical domain of quality of life is related to pain, fatigue, and sleepiness perceptions; these symptoms influence the daily lives of correctional officers due to the work overload caused by excessive work tasks and a reduced number of professionals. In addition, a constantly tense prison climate due to the risk of escapes and physical conflicts requires constant vigilance by these professionals, which could influence their sleeping habits inside and outside the correctional facilities.^2,4,13,26^

On the other hand, the psychological domain comprises aspects such as self-esteem, appearance, as well as positive and negative feelings and their effects on quality of life. Negative feelings are present in the daily lives of correctional officers, since the environment of correctional facilities is psychologically demanding and stressful. Fear, anxiety, and insecurity cross their lives and behaviors inside and outside their work environment.^4,13,26^

Finally, in this study we observed a correlation between quality of life and burnout, since higher burnout levels (in all dimensions) were related to lower quality of life scores, mainly in the physical and psychological health domains. These findings were in line with those of Grensman et al.,^27^ who compared the quality of life of workers that were on leave due to a burnout diagnosis with that of healthy and active workers. Their results indicated that individuals with burnout presented very low quality of life scores when compared to healthy people.

## CONCLUSIONS

The correctional officers interviewed in this study presented moderate burnout levels that could be related to signs of physical and emotional exhaustion, as well as attitudes of insensitivity and severity in interpersonal relationships. Nevertheless, most officers were satisfied with their work, which was possibly related to their reasons for choosing this profession (financial aspects were considered more important than other factors). We also verified that burnout directly interferes with the quality of life of these professionals.

Regarding symptoms of burnout and a decrease in quality of life identified in correctional officers, we highlight the importance of health promotion measures for these workers. In this regard, new studies are necessary for developing strategies that provide better quality of life and protect workers’ health.
